# A Dyciandiamine-Based Methacrylate-Epoxy Dual-Cure Blend-System for Stereolithography

**DOI:** 10.3390/polym13183139

**Published:** 2021-09-17

**Authors:** Manuel Romeis, Dietmar Drummer

**Affiliations:** Institute of Polymer Technology (LKT), Friedrich-Alexander-University Erlangen-Nuremberg, Am Weichselgarten 9, 91058 Erlangen, Germany; dietmar.drummer@fau.de

**Keywords:** dual-cure, stereolithography (SLA), additive manufacturing (AM)

## Abstract

In this research, an epoxy-based dual-cure system is developed and characterized for SLA additive manufacturing. Dual-cure systems consist of UV-curable acrylates and thermal active components. The second curing step offers an additional degree of freedom to design specific material properties. In this study, a blend of varying concentrations of an epoxy/curing agent mix, respectively, DGEBA, DICY and photocurable methacrylate, was used to create a material that is printable in the SLA process into a UV-cured or green part and subsequently thermally cured to achieve superior thermal and mechanical properties. Calorimetric measurements were performed to determine the reactivity of the thermal reaction at different concentrations of epoxy. The fully cured specimens were tested in mechanical and dynamic mechanical measurements, and the results showed a significant improvement in tensile stress and glass transition temperature with rising epoxy concentrations. Fractured surfaces from tensile testing were investigated to further characterize the failure of tested samples, and thermal degradation was determined in TGA measurements, which showed no significant changes with an increasing epoxy concentration.

## 1. Introduction

Stereolithography (SLA) is the oldest additive manufacturing (AM) technique and is based on the generation of three-dimensional parts by the subsequent addition of single layers onto each other, using a photochemical reaction of monomers [1]. This layer-wise and tool-less process, which all AM-techniques have in common, makes it possible to produce complex parts right from the computer-aided-design (CAD) model [2]. SLA printers typically use liquid UV-resins, which cure due to an exothermal chemical reaction initiated by UV-light. The photoreaction leads to the gelification and vitrification of the UV-resin, which means a transition from the formerly liquid state to a solid part [3]. The utilization of the photochemical reaction leads to many advantages compared to other AM-techniques, such as a high production speed paired with high surface quality and the possibility of details in the range of a few microns. Unfortunately, the chemical structure of the UV-cured parts comes along with some significant drawbacks. The main disadvantages are poor mechanical properties and a low heat deflection temperature [4]. Since the single layers are not fully cured, to achieve a chemical connection between each other during the printing process, the manufactured parts require a UV-post-curing treatment. Indeed, achieving a fully cured part during that post-curing treatment is often impossible due to the reaction inhibition induced by contact with oxygen and the limited curing depth [5,6,7]. Dual-cure systems consist of two individual curing steps and represent the key for advancing the pure acrylate systems’ poor mechanical and thermal properties. The dual-cure process achieves this by solving the partially chemical conversion of acrylate groups with a following thermal post-curing-step that is not inhibited by oxygen. Furthermore, an aimed regulation of temperature during radical polymerization can control side reactions and, therefore, the complexity of the polymer network [8]. This style of curing opens up entirely new possibilities for resin-based additive manufacturing.

**Figure 1:**
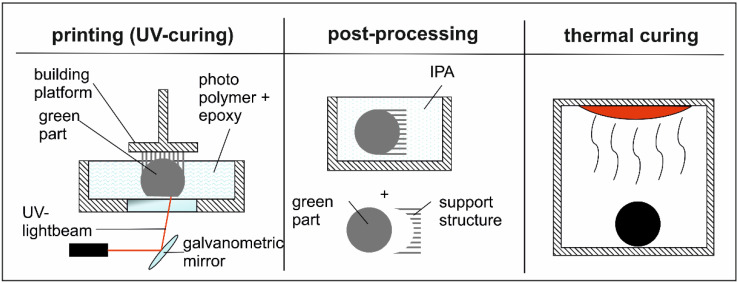
Overview of a dual-cure printing processing.

Different stage-curing epoxies are already a common way to produce epoxy-based prepregs for subsequent manufacturing processes. For this application, the already gelificated epoxy is undercooled to stop a further curing process [9]. Dual-cure systems are a more innovative approach and are, therefore, already utilized in many applications reaching over films, adhesives, and mainly in the coating industry [10,11]. They function via a combination of two curing steps, which can proceed parallel or subsequently to build a so-called interpenetrating network (IPN). As a result, this network structure offers superior properties compared to the individual properties of the single components of the dual-cure material [12]. In that way, dual-cure systems can combine the outstanding advantages of UV-curing systems, such as a fast reaction speed at room temperature combined with a low amount of energy and cost [13] with the favorable properties of the second component. In additive manufacturing, the usage of one-part dual-cure systems based on latent curing agents provides the opportunity to keep the state of the liquid resin processable even for the duration of long print jobs. This process stability of the resin is achieved by inhibiting a premature reaction of the curing agent, which would lead to the unwanted increase in viscosity. Figure 1 shows the schematic printing process with dual-cure materials and the subsequent post-processing steps.

In the field of dual-cure systems for use in additive manufacturing, different promising approaches have already been pursued. Next to the common pure acrylate systems, many resin systems for SLA are based on the usage of the epoxy acrylates [14] or linking elements, which contain both an epoxy and an acrylate group, such as, e.g., 4-vinyl-1-cyclohexene 1,2-epoxide [15] or glycidyl methacrylate (GMT) [16]. While these systems use a dual-cure approach, the usage of these linking elements or acrylated epoxies is problematic as it results in low mechanical properties and weak light aging resistance [17] next to typical UV-acrylate problems such as the inhibition by oxygen. A dual-cure system using a second thermal curing step can advance these problems. In this field, the usage of urethanes as the thermally cured component is studied, mainly. Velankar [18] invented a system of urethane materials via deblocking chemistry, which Carbon3D patented [19] for usage in digital light synthesis (DLS). Different studies were performed on that topic [20,21]. Although approaches have been made for Acrylate/Epoxy dual-cure systems for direct ink writing (DIW) [22,23], DLS [24,25,26] and DLP [27] studies for the SLA process are lacking. Furthermore, recent studies rather focus on improving thermal properties than on mechanical properties. In particular, the usage of an epoxy as a thermal cured component in the dual-cure system offers a wide range of possible part properties due to the almost unlimited range of combinations. In order to compete with established AM techniques such as SLS, FDM and pure acrylate-based SLA, a wider range of material systems has to be developed and further investigated.

In this study, a latent curing methacrylate/epoxy blend is prepared and printed. The aim is to generate a latent curing material system to achieve superior mechanical properties in combination with high thermal stability compared to a pure acrylate system. Due to their good mechanical properties, chemical and electrical resistance and thermal properties, epoxies find usage in many applications over coatings, adhesives, construction, composites and electronics materials [28,29,30], making them a suitable component to reinforce the acrylate system. The used dicyandiamide (DICY) hardener offers materials an exceptional high glass transition temperature, mechanical properties and strong resistance to acids and typical solvents while having a long shelf life at room temperature [31]. The enhanced shelf-life secures the remaining resin to reduce the production of waste, which is a common problem in the processing and recycling of epoxies. These characteristics of this epoxy/hardener combination make it possible to achieve the desired process and part property profiles.

## 2. Materials and Methods

### 2.1. Material

**Figure 2:**
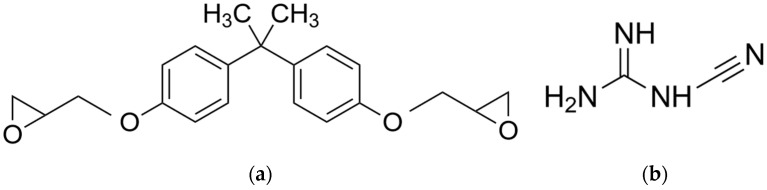
Chemical structure of (**a**) DGEBA; (**b**) DICY.

The material contains two different resin systems, which were mixed in varying ratios. For the UV-resin, the commercial “Clear V4” (further referred to as “Clear” or “methacrylate”) resin of Formlabs (Formlabs Inc., Somerville, MA, USA) is used, consisting of a mixture of 75–90% methacrylated oligomer, 25–50% methacrylated monomers and <1% diphenyl (2,4,6 trimethylbenzoyl) phosphine oxide (TMDPO) as photoinitiator. For the epoxy EPILOX A 18-00 by Leuna Harze (LEUNA-Harze GmbH, Leuna, Germany), a diglycidyl ether of bisphenol A (DGEBA) with an epoxy equivalent weight of 175–185 g is used (Figure 2a). Regarding the curing agent Amicure CG 1200, a latent curing agent based on dicyandiamide (Figure 2b) modified with 0.5% inert flow control additive was used, which was kindly donated by Evonik Industries AG (Evonik Industries AG, Essen, Germany).

In the first step, the epoxy resin was prepared. A total of 100 phr of EPILOX A 18-00 were preheated to 60 °C to lower the viscosity and ensure a homogenous dispersion of the DICY. Next, 7 phr of Amicure were added and mixed using a mechanical stirrer for 10 min at a rotation speed of 200 rpm. This epoxy-curing-agent mixture will further be referred to as epoxy. After the mixture was cooled to 40 °C, a specified amount (Table 1) of Clear resin, further referred as Clear, was added and mixed in a centrifugal mixer for 2 min, at a rotation speed of 2000 rpm until a homogeneous liquid developed. Material combinations with higher epoxy concentrations were also prepared, but they were not able to solidify in the printing process; therefore, they are not listed.

The mixture was degassed in a vacuum chamber with a pressure of 0.03 bar for 5 min to prevent oxygen-induced inhibition during UV-curing and reduction in mechanical properties due to voids.

### 2.2. Printing Process and Thermal Post-Curing

**Figure 3:**
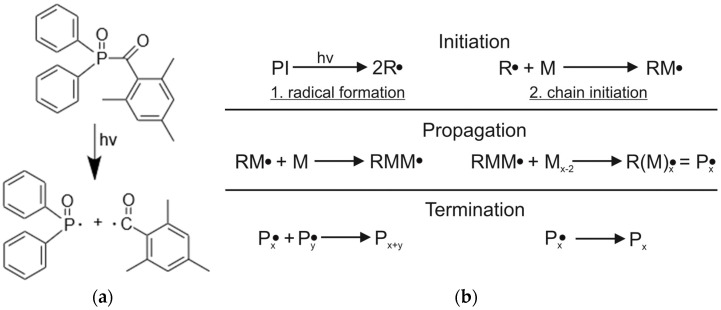
Curing process during printing by (**a**) formation of radicals from TMDPO and (**b**) radical photopolymerization [32,33].

A Form 2 SLA-printer (Formlabs Inc., Somerville, MA, USA) was used for the printing process with the designated presettings for the Clear V4 resin. The printed specimens are tensile bars (DIN EN ISO 527-2; Type 1BA; scaling 1:2) and DMA testing samples (DIN EN ISO 6721, scaling 1:1). For both types of testing specimens, support structures were used with a complete raft, a density of 1.0, and a diameter of the connection point of 0.6 mm. A layer height of 0.1 mm was chosen. After printing the samples, they were first spun to remove the remaining resin and afterward, washed in IPA (concentration of 99.5%) in two stages, with each stage lasting 30 s. After the IPA cleaning, the green parts were carefully dried with pressurized air to avoid the solvent from damaging the still sensitive parts. During the printing process, the photoinitiator (PI) of the Clear resin, TMDPO, respectively, forms radicals subsequently to UV-irradiation (Figure 3a) [32].

These free radicals (R) initiate a chain polymerization with the methacrylated monomers and oligomers (M) to form new radicals (RM●) repeatedly until, after a certain chain length a Polymer (P) of the length x is formed. This reaction occurs until a termination reaction occurs (Figure 3b) [33,34,35]. The complexity of the resulting polymer network is dependent on the functionality of the used monomers and oligomers.

**Figure 4:**
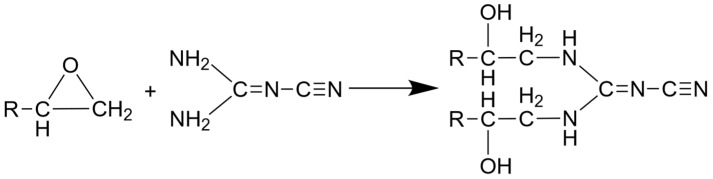
Ring opening reaction of epoxy group and primary amine of DICY (after [30]).

For the thermal post-curing step, a forced convection oven type DKN612C (Yamato Scientific Co., Ltd., Tokyo, Japan) was used with a two-stage heating program. In the first stage, the parts were heated up 165 °C and pre-cured for 1 h and in the second stage, cured at 180 °C for 2 h. The temperature and time for the second stage was gathered from the Amicure CG 1200 datasheet. The first stage was added since pretests showed that heating up to 180 °C in only one step leads to a reaction that is too high, which induced cracking of parts. Exemplary DSC measurements were performed after thermal curing in the oven to secure complete curing. They showed no further curing reactions, which states the oven cycle was sufficient. The curing of DGEBA/DICY happens to be a complex process where multiple different reactions can occur. The essential reactions are, dependent on the temperature and the composition of the resin, a ring opening addition reaction of the epoxy group and the primary and secondary amin of the DICY, the addition of hydroxyl to the oxirane functionality or also called etherification of epoxy group with the hydroxyl group, homopolymerization of epoxy groups and an addition reaction of the nitrile group [30,31]. Since the used epoxy system contains a stochimetric content of DICY and curing happens at low temperature, the main reaction to expect is the ring opening reaction of the epoxy group and the primary amine, shown in Figure 4 [30].

### 2.3. Methods

#### 2.3.1. Differential Scanning Calorimetry (DSC)

Differential Scanning Calorimetry measurements were performed to ensure and identify the intensity of the exothermic reaction occurring between the Amicure curing agent and the DGEBA. For the DSC measurement, a type Jupiter STA 449 F3 DSC device (Netzsch Holding, Selb, Germany) was used. The samples of the Clear/epoxy blends for DSC measurement were cut from UV-cured solid specimen. As a reference for this study, a liquid 100% epoxy concentration sample (100/0) and a 0% epoxy concentration sample (0/100) were added to the measurement, The heat flow of the sample was continuously measured during the heating from 50 to 200 °C, using a heating rate of 5 K/min. For the measurement, an open 85-microliter aluminum crucible was used with a weight of 19 ± 0.5 mg and a sample weight of 8 ± 1 mg. The measurements were performed under nitrogen atmosphere.

#### 2.3.2. Thermogravimetric Analysis (TGA)

Thermogravimetric analysis was performed to determine whether there is an influence of the epoxy on the beginning and speed of material degradation. The TGA measurements were conducted on a type Jupiter STA 449 F3 TGA device (Netzsch Holding, Selb, Germany). The samples were prepared in a similar way to the DSC samples. For the measurement, small samples were heated to 500 °C with a heating rate of 5 K/min, while the loss of mass was determined by constant weighing of the sample. For the measurement an open 85-microliter aluminum crucible was used with a weight of 19 ± 0.5 mg and a sample weight of 8 ± 1 mg. The measurements were performed under nitrogen atmosphere.

#### 2.3.3. Tensile Testing

The samples were stored before the tensile testing at standard climate (T: 23 °C; rel. hum.: 50%) for a minimum of 48 h. Subsequently, the samples were tested with a type 1465 universal testing machine (Zwick Roell GmbH & Co. KG, Ulm, Germany) according to DIN EN ISO 527: 2012, to measure the tensile modulus E_D_, the tensile strength σ_max_ and the elongation at break ε_r_. The testing speed was set to 2.5 mm/min with a pre-force of 0.5 N. The tensile modulus was determined with an extensometer.

#### 2.3.4. Dynamic Mechanical Testing (DMA)

Dynamic mechanical testing (DMA) was used to measure the storage modulus E’, loss modulus E’’ and tan(δ) to determine the glass transition temperature T_g_ of the prepared samples in dependency of the used epoxy proportion. Measurements were performed on a type RSA G-2 DMA measurement device (TA Instruments Inc., New Castle, DE, USA). The load was introduced by torsion with a frequency of 1 Hz and a strain of 0.02%. While keeping mechanical stress constant, a heat ramp of 2 K/min was used, beginning at −60 to 200 °C. The determination of T_g_ was conducted by evaluating the peak of tan(δ).

#### 2.3.5. Microscopy

The fractured surfaces of the samples obtained from the tensile testing were inspected with a scanning electron microscope type Gemini Ultra-Plus (Carl Zeiss AG, Oberkochen, Germany) with a voltage of 10 kV, varying working distance (WD) and a magnification of 22, 50 and 100.

## 3. Results and Discussion

### 3.1. DSC

**Figure 5:**
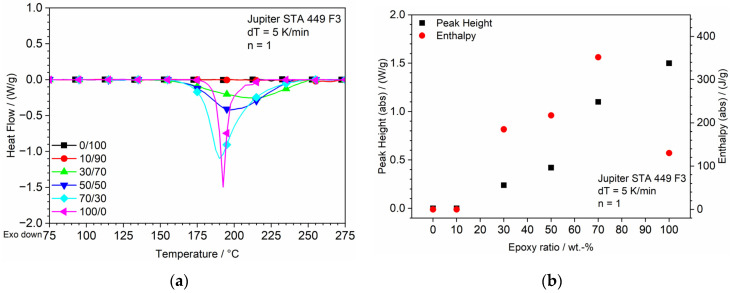
DSC of blends for increasing temperature; (**a**) Heat flow dependent on epoxy concentration; (**b**) Peak analysis of DSC measurement.

DSC measurement is a reliable tool for measuring and visualizing the exothermic reaction of epoxy thermosets. The results of the measurement are shown in Figure 5. The heat flow of the 100/0 curve shown in Figure 5a pictures a strong exothermic peak at approx. 192 °C, beginning at approx. 180 °C, resembling the reaction of the curing agent with DGEBA. For the pure Clear sample (0/100) and a small concentration of DGEBA within sample 10/90, this reaction-peak at this defined temperature is entirely missing, which leads to the assumption that for this low amount of epoxy/curing agent, the reactive groups are not able to react with each other. The absence of a reaction is most certainly based on the lack of mobility of the system and, therefore, spatial separation of the reactants. A second reason for the absence of the reaction-peaks is an increasing blocking effect of the surrounding Clear resin with a low epoxy amount.

The missing peak should also affect the results of the mechanical and thermal properties. The curves of the 30/70, 50/50 and 70/30 samples show the same reaction peak with an increasing intensity for a higher epoxy concentration and a shift to lower temperatures of the peak itself, while the onset of the peak remains at 150 °C. The reason for the increase in peak intensity is that with a higher absolute number of reactive groups, a higher share of reactions can also possibly happen. The shift to lower temperatures combined with the increase in of the gradient of the graphs for higher epoxy concentrations indicates a faster and more intense reaction. This shift can be explained by the auto-accelerating effect of the exothermic DGEBA/DICY-reaction [31]. The pure epoxy sample displays a later start of the reaction at approx. 175 °C paired with the steepest gradient of the graph. This effect will be further explained after the discussion of the peak analysis shown in Figure 5b. The peak height shows (Figure 5b) an increase with a rising epoxy concentration after being null for the pure Clear and the 10/90 sample, which underlines the increasing reaction speed and intensity due to the increasing probability of a reaction. Regarding the evaluation of the enthalpy, different assumptions can be made. At first, the increase in the epoxy concentration leads to an enhanced enthalpy, whereas it drops drastically for the pure epoxy sample. Reasons for that effect and the onset at an elevated temperature within the 100/0 sample could possibly arise from phase separation and the sedimentation of the curing agent due to the temperature-induced high decrease in viscosity before the gelation could happen. Therefore, the intensity of the reaction is higher but due to the spatial different concentration of the curing agent and, therefore, the overstochiometric ratio, only a certain amount of curing agent is able to react because of the limited epoxy groups. Another possible reason for this effect could be a delayed dissolution of the DICY hardener in the DGEBA, which is essential for the start of the reaction. In the case of the epoxy/Clear blends, the curing agent is already bound in the material and equally dispersed, which could possibly ease the dissolving process.

### 3.2. Tensile Testing

The tensile properties of the Clear/epoxy blend, depending on the epoxy concentration, are summarized Table 2. It can be seen that an increasing concentration of epoxy leads to a rise in elastic modulus and tensile stress and an initial decrease and subsequent increase in the elongation at break compared to the pure Clear resin.

**Figure 6:**
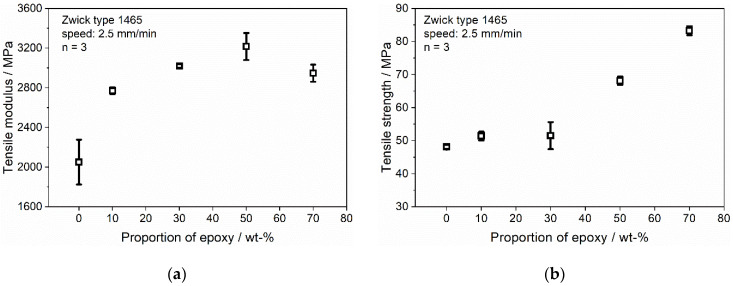
Effect of epoxy concentration on the tensile properties of the Clear/epoxy blend; (**a**) tensile modulus; (**b**) tensile strength.

The variation of the tensile modulus with different concentrations of epoxy is shown in Figure 6a. The tensile modulus shows a logarithmic increase until a concentration of 50% epoxy and slightly decreases to 70%. Although no reaction is detectable in the DSC measurement for the 10/90 sample, the addition of the epoxy system leads to an increase in the tensile modulus. A possible reason for this increase in stiffness could arrive from the incorporation of microimperfections due to the integration of the epoxy. These microimperfections can cause local spikes in stress and lead to an increase in the tensile modulus, similar to the integration of glass fillers. For the 30/70 sample, an epoxy network could be established that leads to an increase in the tensile modulus. The increasing epoxy network leads to an increase in tensile modulus until 50% epoxy content. The decrease in the tensile modulus at 70% epoxy content could possibly arise from the fact that at this concentration, the epoxy structure becomes dominant with its mechanical properties. Therefore, with less Clear resin, the induced microimperfections are also reduced, which leads to a decrease in stiffness.

**Figure 7:**
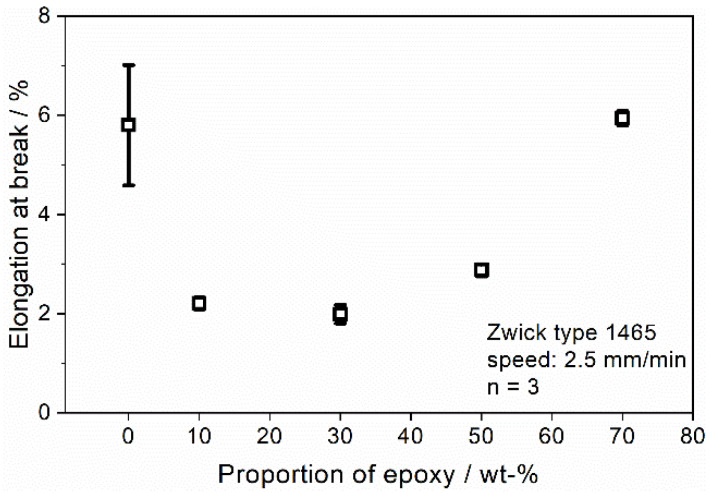
Effect of epoxy concentration on elongation at break of the Clear/epoxy blend.

Figure 6b shows the effect of the epoxy concentration on the tensile stress at break. For low amounts of epoxy, the tensile strength remains at a constant level; therefore, the addition of epoxy at low concentrations shows no influence on the tensile strength of the samples. Although a reaction of the DICY and DGEBA is shown at 30%, no increase in the tensile strength can be observed. The graph’s course indicates that there is a critical level of concentration where the epoxy can build up a connecting structure that is not dominated by the Clear resin. More than a 30% epoxy amount leads to a linear increase in the tensile stress until it climaxes for the maximum concentration of epoxy of 70% in this experiment at a value of 83.27 MPa, which is a 73.1% increase compared to the 0/100 sample and a 62.0% increase compared to the 10/90 sample. The effect of weak properties at low amounts of epoxy until the existence of a corresponding network can also be observed in the graph for the elongation at break (Figure 7). With the addition of epoxy at low concentrations (10 and 30%), there is a considerable decrease in the elongation at break to roughly one third of the initial value. It shows that although a reaction occurred for the 30/70 sample, the resulting epoxy network is not able to enforce the mechanical properties at that concentration. The minimum of the peak is in the area of 30% epoxy concentration. After increasing the amount over that critical level, the epoxy seems to be able to form a coherent polymer structure until its peak at 70% epoxy concentration, which is in the same range as the pure Clear resin.

### 3.3. Microscopy

**Figure 8:**
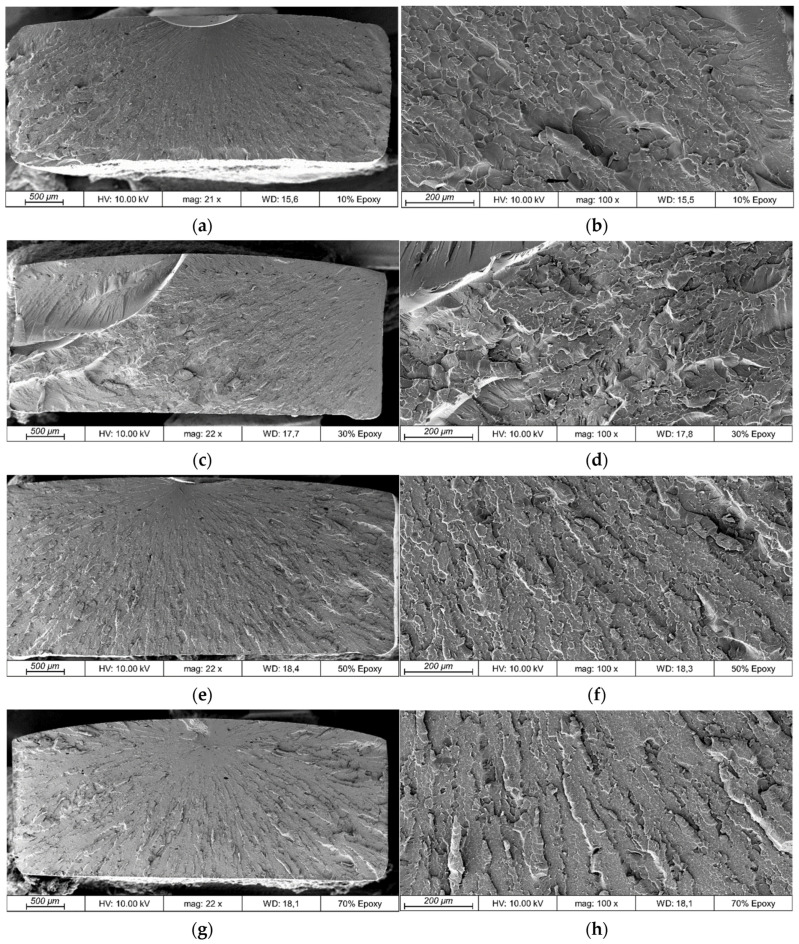
Fracture surface of mechanical testing specimens for different concentrations of epoxy: (**a**) 10/90 21× magnification (mag.); (**b**) 10/90 100× mag.; (**c**) 30/70 22× mag.; (**d**) 30/70 100× mag.; (**e**) 50/50 22× mag.; (**f**) 50/50 100× mag.; (**g**) 70/30 22× mag.; (**h**) 70/30 100× mag.

The examination of the fracture surfaces shown in Figure 8 can give more information on the material behavior during and right before the point of breaking.

For all the different epoxy concentrations, minor defects in the form of voids are identifiable. The reasons for this phenomenon could be insufficient gas evaporation during the degassing process due to the relatively high viscosity of the resin mixture or the consequences of shrinkage and, therefore, residual stress during thermal curing, which is typical for epoxy [36]. Since there is no significant trend for the number of pores given by this small reference group of one sample of each epoxy combination, the initial assumption is that the existence of small air pockets in the resin during the printing leads to the voids in the cured material. However, to give a final explanation, further research on that topic will need to be conducted in the future.

The structure of the fracture surface shows three areas, which is typical for an epoxy fracture. In particular, the zones are the flat mirror zone, the transition zone, characterized by increasing surface roughness, and the final propagation zone [37]. The 10/90 sample shows only a small to nonexistent mirror area and has a broad transition zone beginning right at the fracture ignition point. With an increasing epoxy concentration up to the 70/30 sample, the mirror zone becomes more distinct and larger. This change in the shape of the fracture surface is, combined with the course of the stress–strain graphs, a clear indicator of increasing ductile breaking behavior with an increasing epoxy concentration.

### 3.4. Temperature-Dependent Stability

#### 3.4.1. Dynamical Mechanical Testing

**Figure 9:**
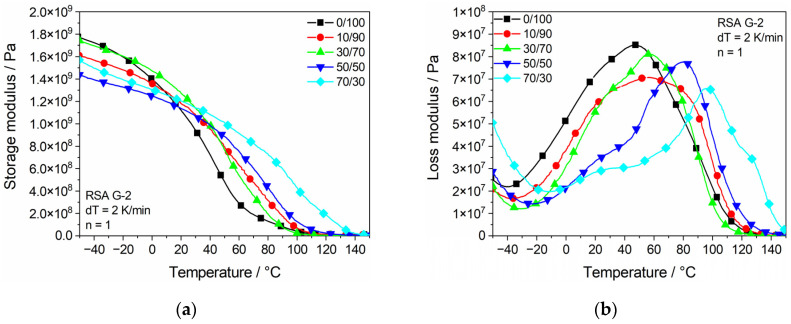
Dynamic mechanical properties dependent on epoxy concentration and temperature: (**a**) storage modulus; (**b**) tan(δ).

Figure 9 shows the storage and loss modulus for different epoxy concentrations for a linear increase in temperature. With a rising epoxy concentration, the storage modulus, shown in Figure 9a, is sinking at temperatures below 0 °C. Although the level is lower at a minus temperature, an increase in the epoxy concentration also leads to a later and slower decrease in the storage modulus with an increasing temperature and, therefore, to a later softening of the material.

**Figure 10:**
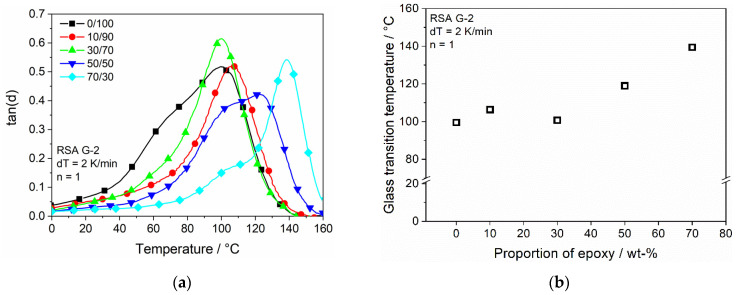
Dynamic mechanical properties dependent on epoxy concentration and temperature: (**a**) tan(δ); (**b**) Tg (from tan(δ)) dependent of epoxy concentration.

As shown in Figure 9b, the loss modulus shows a shift of the global peak to higher temperatures with an increasing epoxy concentration. While being in the same area for low concentrations, it experiences a clear increase after exceeding a concentration of 30% epoxy. This change in behavior matches with the critical amount of 30% shown in the mechanical analysis for the epoxy to form a sufficient network. At approx. 30 °C, a local peak is existent for both the 50/50 and the 70/30 samples. The temperature of these peaks matches the global peak temperature of the 0/100, 10/90 and 30/70 samples, which leads to the assumption that the local peak resembles the transition temperature of the remaining Clear resin in the mixture. The same trend occurs for tan(δ) of the samples depending on the epoxy concentration and temperature, shown in Figure 10a. While the global peak remains around the same temperature of approx. 100 °C for samples 0/100, 10/90 and 30/70, it increases gradually for 50/50 and 70/30.

For the 50/50 sample, the graph of tan(δ) results in a broad peak while being distinct for the 70/30 sample. The graphs for the high epoxy concentrations, on the other hand, again show the double peak structure since the broad peak of 50/50 is probably a superimposition of two single peaks. This double peak supports the initial assumption of the existence of two T_g_, which is traced back to the two different materials in the mixture. Figure 10b pictures the temperature of the global peak and shows an increase with the rising epoxy concentration from 99.5 to 139.2 °C; therefore, in conclusion, the addition of high amounts of epoxy into the dual-cure system leads to better temperature stability.

#### 3.4.2. Thermographic Analysis

**Figure 11:**
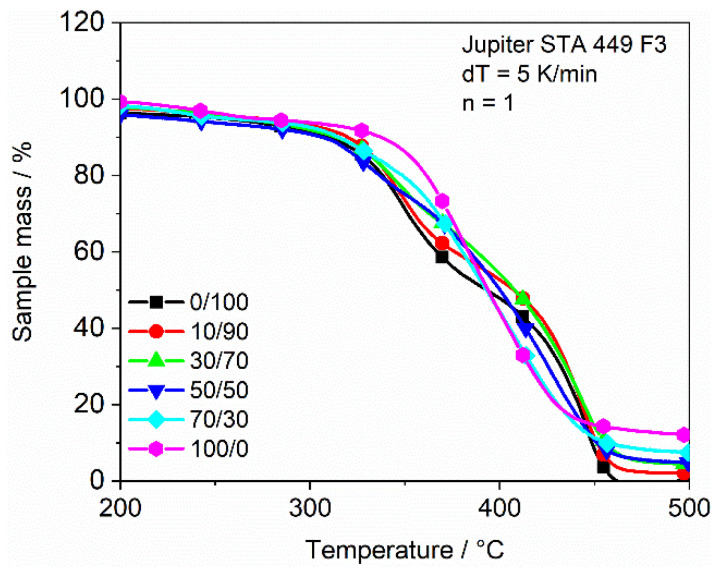
Weight loss with increasing temperature for variable epoxy concentrations.

The results of the TGA measurements, shown in Figure 11, picture the start of the thermal degradation process of the material. For all the epoxy/Clear blends, the mass starts to decrease rapidly after approx. 290 °C; therefore, the addition of epoxy appears to have no significant influence on the start of the thermal degradation until the maximum concentration conducted in this research.

Only the reference sample of 100% epoxy is mostly stable until approx. 350 °C before it also drops rapidly. It stands out that the 0/100 and the 10/90 sample both show two clear steps in the material degradation, probably due to the two different types of methacrylate used in the Clear resin. This two-step degradation is still present for the 30/70 sample but absent for higher epoxy concentrations. With an increasing epoxy concentration, the double peak structure morphs into a local linear decrease in mass, probably due to the superimposition of the two degradation processes of Clear resin and epoxy composition. The 100/0 sample shows only one clear degradation step. Besides the change to only one degradation step with the increasing epoxy content, the speed of the degradation process is also decelerated until the crossover with the graph of the 100/0 sample. After this crossover, the speed of degradation is accelerated with an increasing epoxy contend. It stands out that the samples show different residuals that are proportional to their related content of epoxy. While the 0/100 sample shows no residuals, they are increasing until the 100/0 pure epoxy sample.

## 4. Conclusions

UV-resin blended with different amounts of epoxy, DGEBA/DICY, respectively, for resin-based additive manufacturing was prepared, successfully printed and investigated. With the used printing-system and the standard parameter set, a concentration of a maximum of 70 wt.-% of epoxy was achievable. Higher concentrations led to an insufficient layer adhesion followed by misprints. However, it was found that the first layers, the so-called base layers, were able to be printed at higher concentrations. The printer automatically uses higher exposure settings for these early layers to achieve a good layer adhesion to the building platform. Consequently, adjusting the exposure settings for the following layers should also allow higher concentrations to be printed. DSC measurements showed that a reaction is prevented for low concentrations of epoxy, probably by a separation of reactive groups due to the poor mobility of the system and the low absolute number of reactive groups. This effect was also present in the analysis of mechanical and thermomechanical properties, where no significant changes were found for low concentrations up to 30 wt.-%. After a critical concentration of 50% epoxy, a reaction of DGEBA and DICY occurred, which led to changes in the material properties of the manufactured samples. While not significantly changing the elongation at break, the addition of 70% epoxy led to an increase in the tensile stress from 48 to 83 MPa and a shift of glass transition temperature from 90 to 140 °C compared to the benchmark pure UV-resin parts considered under perfect manufacturing conditions. The structure of the fracture surface also resembled a shift to a more ductile behavior. For thermal degradation, no significant changes could be measured. Since the aim of this work was to achieve an increase in mechanical and thermal properties, the results can be stated as successful. However, the samples showed a critical concentration after which an exponential increase in the tensile stress, elongation at break and glass transition temperature occurred. An epoxy concentration above 70% has a high chance of leading to another significant increase in the mechanical properties, which will be studied in the following investigations.

## Figures and Tables

**Table 1:** Formulation of epoxy blend samples used in this study.

Sample No.	Sample Label	DGEBA/DICY (Epoxy) (wt.-%)	Clear (Methacrylate) (wt.-%)
1	0/100	0	100
2	10/90	10	90
3	30/70	30	70
4	50/50	50	50
5	70/30	70	30
6 *	100/0	100	0

* Only used as a reference for DSC and TGA measurement; cured in DSC process.

**Table 2:** Tensile properties of the epoxy/Clear mixture.

Properties	Amount of Epoxy (wt-%)				
	0	10	30	50	70
Elastic modulus, E_D_ (MPa)	2050 ± 226	2770 ± 34	3020 ± 20	3217 ± 36	2947 ± 86
Tensile strength, σ_max_ (MPa)	48.1 ± 1.3	51.4 ± 1.3	51.5 ± 4.0	68.1 ± 1.2	83.3 ± 1.4
Elongation at break, ε_r_ (%)	5.8 ± 1.2	2.2 ± 0.1	2.0 ± 0.2	2.9 ± 0.1	5.9 ± 0.1

n = 3.

## Data Availability

The data presented in this study are available on request from the corresponding author.
